# Smoking, heavy drinking, physical inactivity, and obesity among middle-aged and older adults in China: cross-sectional findings from the baseline survey of CHARLS 2011–2012

**DOI:** 10.1186/s12889-020-08625-5

**Published:** 2020-07-06

**Authors:** Lijie Ding, Yajun Liang, Edwin C. K. Tan, Yin Hu, Chi Zhang, Yanxun Liu, Fuzhong Xue, Rui Wang

**Affiliations:** 1 Division of health management, School of sport social science, Shandong Sport University, Room 1415, 10600 Shijidadao Road, Jinan, 250102 China; 2 Department of Public Health Sciences, Karolinska Institutet (KI), Stockholm, Sweden; 3 The University of Sydney, Faculty of Medicine and Health, School of Pharmacy, Sydney, New South Wales Australia; 4 Aging Research Center, Department of Neurobiology, Care Sciences and Society, Karolinska Institutet and Stockholm University, Stockholm, Sweden; 5 Centre for Medicine Use and Safety, Faculty of Pharmacy and Pharmaceutical Sciences, Monash University, Parkville, Australia; 6 Södertälje Hospital, Stockholm, Sweden; 7 Energy Processes, Royal Institute of Technology, Stockholm, Sweden; 8 Department of Biostatistics, School of Public Health, Shandong University, Box 100, 44 Wenhua Xi Road, Jinan, 250012 PO China; 9 The Swedish School of Sport and Health Sciences, GIH, Lidingövägen 1, Box 5626, SE-11486 Stockholm, Sweden; 10 Department of Medicine and Wisconsin Alzheimer’s Disease Research Center, University of Wisconsin School of Medicine and Public Health, Madison, WI USA

**Keywords:** Behavioral risk factors, Public health, Cardiovascular disease prevention, Diabetes, Hypertension, High cholesterol

## Abstract

**Background:**

Prevention and control of cardiometabolic conditions and cardiovascular disease (CVD) in China may contribute to sustainable CVD reduction globally, given the fact that one-fifth of the worldwide population is in China. Knowing the distribution of behavioral risk factors (e.g., smoking and physical inactivity), especially at a national level in China, would be extremely relevant to the field of public health and CVD prevention. The objectives of this study were to investigate the nationwide prevalence of obesity, smoking, heavy drinking, and physical inactivity in Chinese adults, and further explore whether cardiometabolic conditions would modify the distribution of behavioral risk factors.

**Methods:**

This population-based study is based on the China Health and Retirement Longitudinal Study (2011–2012), including 17,302 adults (≥45 years, mean age 59.67 years, female 51.66%) from 25 provinces in China. Data on demographics, lifestyle factors, health status and history of diseases were collected via structured interviews and laboratory tests. Smoking, heavy drinking, obesity, and physical inactivity were defined following standard guidelines. We performed descriptive analysis and logistic regressions in this study.

**Results:**

The overall prevalence of heavy drinking, obesity, current smoking, and physical inactivity among middle-aged and older adults was 7.23% (95% confidence interval 6.53—7.29%), 11.53% (10.43—12.62%), 27.46% (26.30—28.62%), and 44.06% (41.19—46.92%), respectively. The prevalence varied between rural and urban areas as well as among geographic areas, with higher prevalence in the Northern and Northeastern regions. Heavy drinking and obesity were significantly associated with incident hypertension, diabetes, and high cholesterol; while current smoking was significantly associated with incident hypertension. Compared with healthy individuals, participants who self-reported a diagnosis of hypertension, high cholesterol, or diabetes were less likely to smoke currently and drink alcohol heavily, but more likely to be physically inactive and obese.

**Conclusions:**

Among Chinese middle-aged and older adults, the prevalence of behavioral risk factors varies by geographic region. Further effort is required to improve physical activity and fitness for Chinese adults, especially those with cardiometabolic conditions.

## Background

Cardiovascular diseases (CVDs), principally stroke and coronary heart disease, are the leading cause of death worldwide [1]. In China, the morbidity and mortality of CVDs have increased persistently over recent decades [2], and approximately 3 million CVD-related deaths and 230 million CVD cases are detected in China every year [3, 4]. To prevent and better control CVDs, the World Health Organization has categorized cardiovascular risk factors into two major groups, cardiometabolic risk factors (e.g., hypertension, diabetes, and high cholesterol) and behavioral risk factors (e.g., smoking, unhealthy diet, physical inactivity, and harmful alcohol use) [5]. Behavioral risk factors are known as easily modifiable factors and are associated with not only CVDs but also cardiometabolic risk factors [6, 7]. Thus, knowing the distribution of behavioral risk factors, especially at a national level, would be extremely relevant to the field of public health and CVD prevention.

In 2015, a Chinese national statistical report showed that among Chinese adults aged 18 years and over, the overall prevalence of behavioral risk factors ranged from 9.3% for harmful drinking to 71.3% for physical inactivity [8]. However, information on the specific geographic distribution of these behavioral factors in China is lacking. This is critical as certain behavioral risk factors, such as smoking, may vary in prevalence between rural and urban areas [9, 10]. Moreover, given the fact that China has now become an aging society, it would also be important to investigate the pattern of behavioral risk factors with age among older Chinese adults.

Furthermore, the number of Chinese people with CVDs and other cardiometabolic conditions is growing rapidly [11–13]. This is largely due to unhealthy lifestyles, such as being overweight and reduced engagement in physical activity. Improving behavioral risk factors among middle-aged and older adults has potential benefits for preventing both cardiometabolic conditions and CVDs. Middle-aged and older adults who have one or more cardiometabolic conditions are expected to improve their lifestyle behaviors to reduce the risks of CVDs. However, questions remain on whether the associations between behavioral risk factors and cardiometabolic conditions vary by the awareness of health conditions.

Studies on the distribution of a broad range of behavioral risk factors in China, especially at a national level, are scarce. Using the China Health and Retirement Longitudinal Study (CHARLS) of adults aged 45 years and over, we aim to 1) investigate the prevalence of four behavioral-related risk factors (i.e., smoking, heavy drinking, physical inactivity and obesity) by age and sex; 2) verify whether there are geographic variations of behavioral risk factors in China; 3) explore the associations between behavioral risk factors and cardiometabolic conditions.

## Methods

### Study design and population

The detailed design of the CHARLS has been reported previously [14]. In brief, the CHARLS is a nationally representative longitudinal survey of Chinese adults (≥45 years). The baseline survey was conducted between June 2011 and March 2012, and subjects were then followed every 2 years, using a face-to-face computer-assisted personal interview by trained interviewers [14].

In this study, we used data from the baseline survey. Data collection was performed according to four standardized stages. In the first stage, 150 county-level units were randomly chosen with a probability-proportional-to-size sampling technique. In the second stage, administrative villages (rural)/neighborhoods (urban) were used as primary sampling units (PSUs), and 3 PSUs were selected in each county. In the third stage, households with residents aged ≥45 years within each PSU were selected. Finally, for a selected household, one random resident aged ≥45 years was selected as a participant of the survey. If the spouse of the selected resident was ≥45 years, the spouse was also included in the survey. All stages of the sampling were conducted by computer, and the response rate among eligible households was 80.51% (conducted in 10,257 households of 12,740 age-eligible households). Of the final sample of 17,708 individual participants of the interview, 506 were excluded from this study due to age < 45 years or missing information on sex. Finally, 17,302 subjects were included in our analytical sample.

The study was conducted in accordance with the Declaration of Helsinki, and the original CHARLS was approved by the Ethical Review Committee of Peking University, and all participants signed informed consent at the time of participation.

### Measurements

The information on demographic factors, lifestyle and behavior (e.g., smoking habits), use of medications (e.g., antihypertensive medications), and history of diseases (e.g., hypertension) were collected at baseline. Highest level of school for each participant was obtained, and education level was categorized into three groups: 1) sishu/home school and below (illiterate or did not finish elementary school); 2) elementary school and middle school; and 3) high school and above. Anthropometric measurements included height, weight and waist circumference. Body mass index (BMI) was calculated by dividing weight in kilograms by height in meters squared. Systolic and diastolic blood pressures were measured on the left arm three times with a 45 s interval in a sitting position, and the average of the three measurements was used in this study [15].

The classification of urban/rural area was based on the definition from the National Bureau of Statistics. We classified provinces into seven major regions: 1) South, including Guangdong, Guangxi, and Hainan; 2) North, including Beijing, Tianjin, Hebei, Shanxi, and Inner Mongolia; 3) East, including Shanghai, Shandong, Jiangsu, Anhui, Jiangxi, Zhejiang, and Fujian; 4) Central, including Hubei, Hunan, and Henan; 5) Southwest, including Chongqing, Sichuan, Guizhou, Yunnan, and Tibet; 6) Northwest, including Shaanxi, Gansu, Ningxia, Xinjiang, and Qinghai; and 7) Northeast, including Heilongjiang, Jilin, and Liaoning [16].

### Assessment of behavioral risk factors

Two questions were used to define smoking status (current, former versus never). The first question asked, “if the participant had ever chewed tobacco, smoked a pipe, smoked self-rolled cigarettes, or smoked cigarettes/cigars”. If the participant answered yes to the first question, the following question asked, “if he/she still has the habit or has totally quit”. Participants were classified into the current smoker group if they still used tobacco, and those who had quit smoking were classified into the former smoker group [17]. A standard drink was defined as 13.6 g of ethanol according to Chinese standards, and was equivalent to 375 ml of beer or 118 ml of wine [18]. The total number of drinks for each participant was thus calculated. Heavy drinking was further defined as > 14 drinks/week for males and > 7 drinks/week for females [19]. BMI was categorized into underweight (< 18.5 kg/m^2^), normal weight (18.5 to 23.9 kg/m^2^), overweight (24 to 27.9 kg/m^2^), and obesity (≥28 kg/m^2^) [20]. Only a randomly selected subsample (6900 out of 17,708) of participants received the physical activity questionnaire, to help explain and assess the consistency of the self-reported health status [14]. It is a validated questionnaire collecting frequency, time of vigorous, and moderate-to-light physical activity of participants in a usual week [21, 22]. Physical activity was defined as ≥150 min/week of moderate, or ≥ 75 min/week of vigorous activity, or a combination (≥600 metabolic equivalents [METs])) [23]. For those who did not meet the criteria of physical activity, they were assigned to the physically inactive group.

### Assessment of diabetes, hypertension, and high cholesterol

Hypertension was defined as blood pressure ≥ 140/90 mmHg at baseline or use of antihypertensive medications [24]. Diabetes was defined as fasting plasma glucose ≥7.0 mmol/L (126 mg/dl), 2-h plasma glucose ≥11.1 mmol/L (200 mg/dl), HbA1c concentration ≥ 6.5%, reported history of diagnosed diabetes, or use of anti-diabetic medications [25]. High cholesterol was defined as fasting serum total cholesterol of ≥6.22 mmol/l or use of cholesterol lowering medications [26].

Patients with hypertension, diabetes, and high cholesterol were further divided into a ‘previously aware’ group (those patients who reported a previous diagnosis of hypertension, diabetes, or high cholesterol), and a ‘newly detected’ group (those patients who did not report a diagnosis of hypertension, diabetes, or high cholesterol, but had an increased level of blood pressure, plasma glucose, or cholesterol at baseline examination).

### Statistical analysis

All analyses were weighted to represent the overall Chinese adult population aged ≥45 years, by using individual sample weights to adjust for non-response [27]. We checked the normality of the variables before running the analysis further. Characteristics of participants by living areas were compared by the chi-square test for categorical variables or t-test for the continuous variables (i.e., age). Overall prevalence of current smoking, heavy drinking, physical inactivity, and obesity were calculated. We described the prevalence of individual behavioral risk factors by sex, age, living areas (rural vs. urban), and geographic areas (South, North, East, Central, Southwest, Northwest, and Northeast). To visually illustrate geographical distribution of the behavioral risk factors, we further showed the province-specific prevalence with a density map. We applied DataMap for Excel 5.1.2 to generate the density maps of behavioral risk factors.

To examine the relationship between the behavioral risk factors and cardiometabolic conditions, binary logistic regressions and multinomial logistic regressions were used to estimate the odds ratios (ORs) and 95% confidence intervals (CIs). In the binary logistic regressions, cardiometabolic conditions (yes versus no) were treated as dependent variables and behavioral risk factors were treated as independent variables. To further investigate the association between behavioral risk factors and cardiometabolic conditions, we divided the participants who were with cardiometabolic conditions into two groups: newly detected group and previously aware group. The multinomial logistic regressions were performed to estimate the ORs and 95% CI of newly detected cardiometabolic conditions and previously diagnosed cardiometabolic conditions in relation to behavioral risk factors, respectively.

*P*-values < 0.05 were considered statistically significant. Data analysis was conducted in June to October, 2016. SAS statistical software version 9.4 (SAS Institute Inc., Cary, NC) was applied in this study.

## Results

Table 1 presents characteristics of study participants (*n* = 17,302) by living areas (rural v.s. urban). Overall, the mean age was 59.67 years, 51.66% were female, and 15.40% had an education level of high school and above. The overall prevalence of heavy drinking, obesity, current smoking, and physical inactivity were 7.23% (95% CI 6.53—7.29%), 11.53% (10.43—12.62%), 27.46% (26.30—28.62%), and 44.06% (41.19—46.92%), respectively, and the overall prevalence of hypertension, diabetes, high cholesterol and CVDs were 39.13% (37.55—40.71%), 12.29% (11.44—13.14%), 15.86% (14.82—16.91%), and 13.82% (12.69—14.95%). Compared to people living in urban areas (*n* = 7003), the rural participants (*n* = 10,299) had a lower level of education, were more likely to be current smokers, were less likely to be obese and physically inactive, and had a lower prevalence of hypertension, diabetes, high cholesterol and CVDs (*P* < 0.05). The two groups did not differ in age and alcohol consumption.

**Table 1:** Characteristics of study participants at baseline

	Total (*n* = 17,302)	Urban (*n* = 7003)	Rural (*n* = 10,299)	***P***-value
**Age, year, mean (Standard deviation)**	59.67 (19.71)	59.49 (24.27)	59.86 (19.28)	0.280
**Age groups, n (%)**				0.131
45-	6079 (36.22)	2537 (37.67)	3542 (34.77)	
55-	6439 (34.95)	2558 (34.12)	3881 (35.78)	
65-	3228 (18.02)	1249 (17.26)	1979 (18.78)	
75-	1348 (9.05)	574 (9.27)	774 (8.83)	
85-	208 (1.76)	85 (1.68)	123 (1.84)	
**Female, n (%)**	8881 (51.66)	3679 (52.49)	5202 (50.82)	0.022
**Education level, n (%)**^**a**^				< 0.001
Sishu/home school and below	7801 (42.51)	2272 (30.17)	5539 (54.86	
Elementary school and middle school	7259 (42.10)	3162 (45.24)	4097 (38.94)	
High school and above	2211 (15.40)	1559 (24.59)	652 (6.20)	
**Smoking status, n (%)**^**a**^				< 0.001
Never	10,278 (63.47)	4297 (65.67)	5981 (61.29)	
Former	1410 (9.07)	609 (10.13)	801 (8.03)	
Current	4829 (27.46)	1765 (24.21)	3064 (30.68)	
**Alcohol consumption, n(%)**^**a**^				0.081
Never	11,822 (70.13)	4855 (71.38)	6967 (68.88)	
Former	1040 (5.62)	424 (5.52)	616 (5.72)	
Current but not heavy drinker	2996 (17.03)	1146 (16.64)	1850 (17.41)	
Heavy drinker	1352 (7.23)	519 (6.46)	833 (7.99)	
**BMI category, n (%)**^**a**^				< 0.001
Underweight (< 18.5)	918 (6.81)	227 (4.50)	691 (8.65)	
Normal (18.5–23.9)	7056 (52.01)	2299 (45.58)	4757 (57.12)	
Overweight (24.0–27.9)	3849 (29.66)	1679 (35.00)	2170 (25.41)	
Obesity (≥28)	1501 (11.53)	729 (14.92)	772 (8.82)	
**Physical inactivity, n (%)**^**b**^				< 0.001
No	4034 (55.94)	1330 (45.32)	2704 (65.80)	
Yes	2727 (44.06)	1388 (54.68)	1339 (34.20)	
**Hypertension, n (%)**				0.003
Previously knew	4298 (26.21)	1967 (29.46)	2331 (22.97)	
Newly detected	2225 (12.92)	843 (12.05)	1382 (13.78)	
**High Cholesterol, n (%)**				< 0.001
Previously knew	1579 (10.11)	881 (13.41)	698 (6.81)	
Newly detected	1090 (5.75)	382 (4.45)	708 (7.06)	
**Diabetes, n (%)**				< 0.001
Previously knew	992 (6.16)	562 (8.18)	430 (4.14)	
Newly detected	1107 (6.13)	421 (5.68)	686 (6.58)	
**Cardiovascular diseases, n (%)**	2380 (13.82)	1171 (15.98)	1209 (11.68)	< 0.001

^**a**^There are 31 missing in education, 785 missing in smoking, 92 missing in alcohol consumption, 3978 missing in measuring BMI, 181, 439, 253 and 141 in hypertension, high cholesterol, diabetes and cardiovascular diseases

^b^Physical inactivity was defined in a sub-sample, with total number 6761, of them, 2718 were in urban area, and 4043 were in rural area. BMI: body mass index

**Fig. 1:**
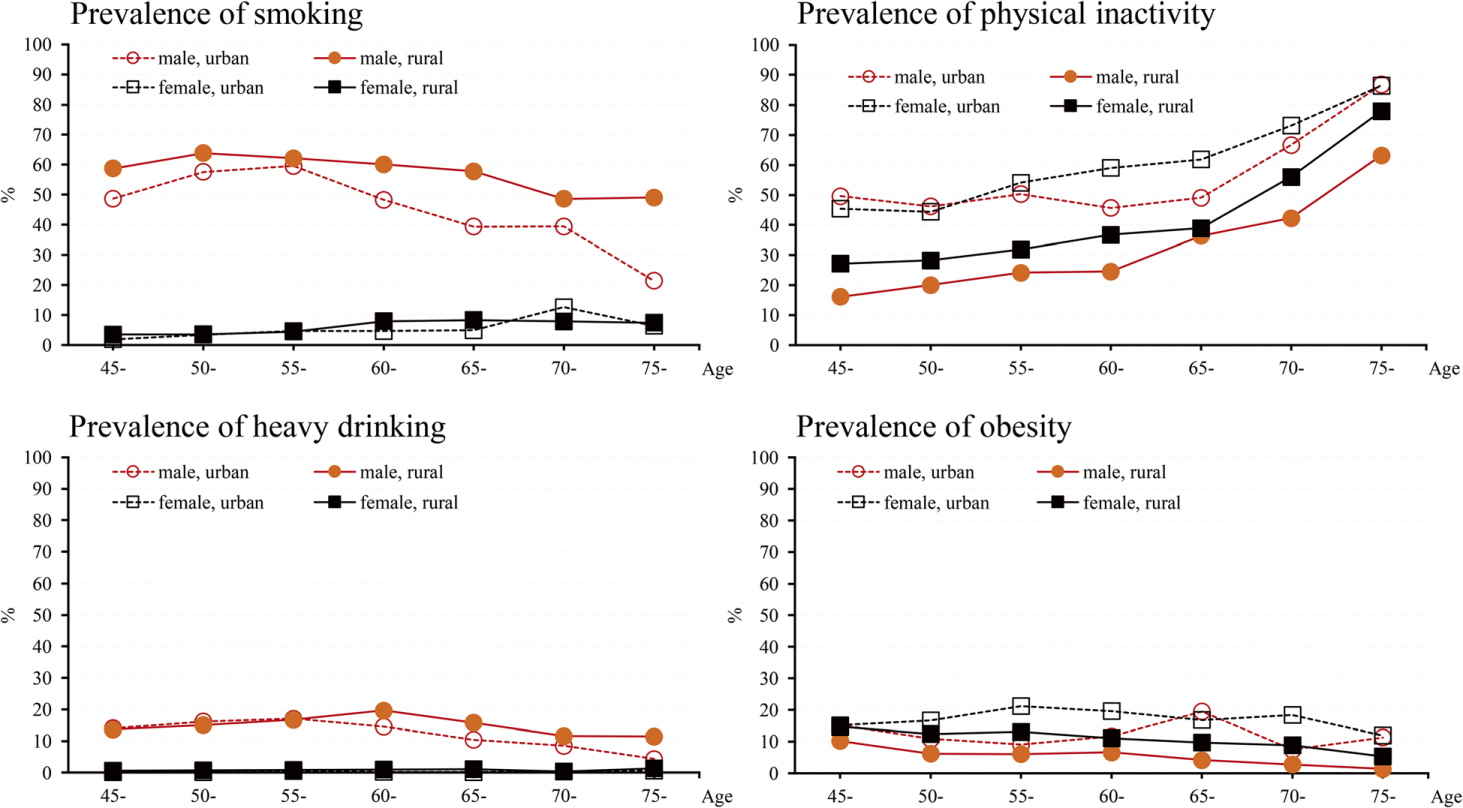
Prevalence of current smoking, heavy drinking, physical inactivity and obesity by age, sex, and areas

The age, sex and urban/rural specific prevalence of current smoking, heavy drinking, physical inactivity and obesity are shown in Fig. 1. Male showed higher prevalence in smoking and heavy drinking than female across all age groups, whereas female showed higher prevalence in physical inactivity and obesity than male. A decreasing trend with age was observed for the prevalence of current smoking in both rural and urban males, and for heavy drinking in urban males from 55 to 65 years, and in rural males from 60 to 70 years. An increasing trend with age was observed in the prevalence of physical inactivity across all participants. The prevalence of obesity was increasing with age in urban males from 45 to 55 years.

The 3 highest regions for the prevalence of smoking were northeast (31.12% [95% CI 29.12—34.13%]), north (29.73% [26.35—33.12%]) and southwest (29.29% [26.77—31.81%]). The 3 highest regions for the prevalence of heavy drinking were southwest (9.67% [8.00—11.34%]), northeast (9.63% [7.31—11.95%]) and east (9.06% [7.83—10.28%]). The 2 highest regions for the prevalence of physical inactivity were northeast (54.91% [46.44—63.37%]) and north (53.07% [47.25—58.9]). The 2 highest regions for the prevalence of obesity were north (19.36% [16.39—22.34%]) and northeast (14.19% [12.08—16.3%]). Detailed information is reported in **Supplementary Table** **1**.

**Fig. 2:**
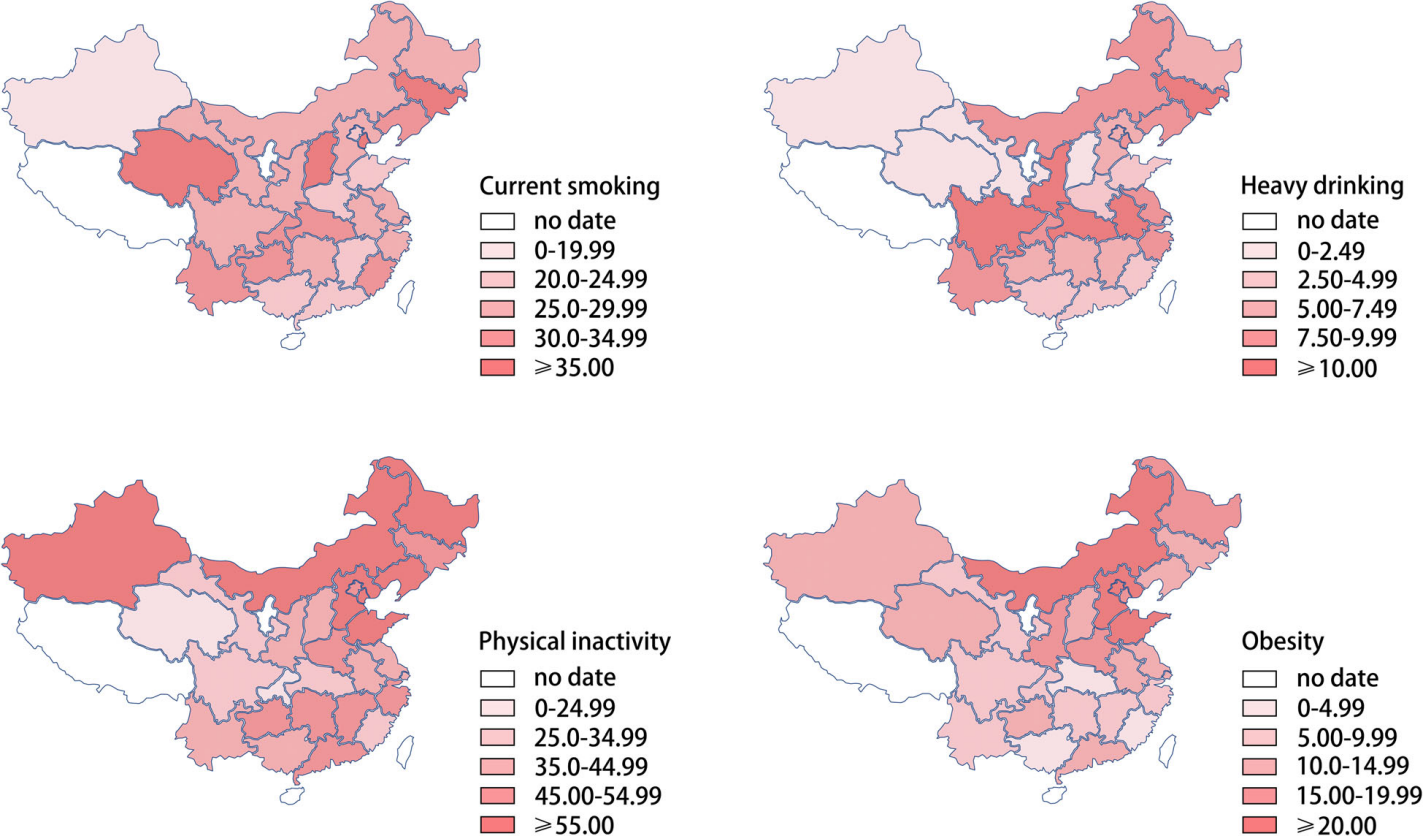
Prevalence of current smoking, heavy drinking, physical inactivity and obesity by provinces. *Notes*. The density maps were generated by DataMap for Excel 5.1.2, using the Chinese geographic map template. An appropriate license from Microsoft has been obtained

Figure 2 further shows the province-specific prevalence of behavioral risk factors. We additionally showed the province-specific prevalence of behavioral risk factors by urban and rural areas (**Supplementary Fig.** **1**), respectively, in the supplement materials.

Among the four behavioral risk factors, physical inactivity and obesity were both significantly associated with higher risk of hypertension, high cholesterol, and diabetes, heavy drinking was only significantly associated with hypertension, whereas smoking was not associated with any of the cardiometabolic conditions (**Supplementary Table** **2**). To better understand the association between behavioral risk factors and metabolic conditions, we divided participants with cardiometabolic conditions into two groups: newly detected group and previously aware group. The results from multinomial logistic regression showed that, compared with healthy subjects (no hypertension, no high cholesterol, or no diabetes), newly detected hypertensive patients were more likely to smoke (OR, 1.34; 95% CI, 1.10—1.64), be heavy drinkers (1.45; 1.17—1.81), and be obese (1.94, 1.51—2.49); newly detected high cholesterol patients and newly detected diabetics were both more likely to be heavy drinkers and obese (Table 2). However, compared with the healthy subjects, patients who were previously aware of their diagnosis (hypertension, high cholesterol, or diabetes) were less likely to be smokers and heavy drinkers, but more likely to be physically inactive and obese.

**Table 2:** Association between behavioral risk factors and cardiometabolic conditions

	Newly detected, Odds ratio (95% confidence interval)			Previously aware, Odds ratio (95% confidence interval)		
	Hypertension	High cholesterol	Diabetes	Hypertension	High cholesterol	Diabetes
Current smoking	1.34(1.10—1.64)^**^	0.99(0.80—1.24)	1.00(0.82—1.23)	0.73(0.63—0.83)^**^	0.73(0.60—0.88)^**^	0.64(0.47—0.86)^**^
Heavy drinking	1.45(1.17—1.81)^**^	1.36(1.04—1.76)^*^	1.30(1.00—1.70)	0.85(0.69—1.04)	0.83(0.64—1.09)	0.61(0.42—0.91)^*^
Physical inactivity	1.20(0.92—1.56)	1.14(0.92—1.43)	0.92(0.70—1.21)	1.31(1.10—1.55)^**^	1.34(1.10—1.64)^**^	1.69(1.26—2.25)^**^
Obesity	1.94(1.51—2.49)^**^	1.32(1.06—1.66)^*^	1.70(1.34—2.16)^**^	4.62(3.95—5.41)^**^	3.25(2.67—3.96)^**^	2.17(1.69—2.79)^**^

*Notes*. Multinomial logistic regression was used to estimate the odds ratio (95% confidence interval) of newly detected and previously aware cardiometabolic conditions in relation to current smoking, heavy drinking, physical inactivity, and obesity. Models were adjusted for age, sex, education and rural/urban area

^*^0.01 < *P* < 0.05; ^**^P < 0.01

The results did not change after excluding participants with CVDs at baseline (**Supplementary Table** **3**).

## Discussion

Our analysis of the CHARLS showed the overall prevalence of heavy smoking (7.23%), obesity (11.53%), current smoking (27.46%), and physical inactivity (44.06%) in adults aged 45 years and over in China. The major findings are: (1) across all age groups, male showed higher prevalence in smoking and heavy drinking, and lower prevalence in physical inactivity and obesity than female; overall, the prevalence of smoking and heavy drinking was decreasing with age, and physical inactivity was increasing with age. (2) The prevalence of physical inactivity and obesity were significantly higher in urban than in rural areas, while the prevalence of current smoking was higher in rural areas. In addition, the prevalence of behavioral risk factors was highly prevalent in northeast and northern areas. (3) Compared to healthy individuals, participants with newly detected cardiometabolic conditions were more likely to smoke, drink alcohol heavily, and to be physically inactive and obese, whilst people who were previously aware of their cardiometabolic conditions were less likely to smoke and drink alcohol heavily, but more likely to be physically inactive and obese.

With our data, we found that the overall prevalence of current smoking and heavy drinking were higher in the Chinese population than in the American population, but the prevalence of obesity and physical inactivity were lower [28–31]. Similar to the findings from the US surveillance data, we also observed that in China, the prevalence of smoking and drinking in male decreased with age since 55–60 years old, and the prevalence of physical inactivity increased with age across all age groups in both male and female [28–31]. Although the Chinese national report (2015) has previously reported the prevalence of behavioral risk factors among adults aged 18 years and over [8], the current study additionally presented the prevalence of those behavioral risk factors in a middle-aged and older population. To our knowledge, this is the first study that investigates the geographic distribution of four comprehensive behavioral risk factors, using nationally representative data of middle-aged and older adults that covers most provinces in China [32, 33].

We observed that urban residents were more likely to be obese and physically inactive than rural residents, whereas rural residents were more likely to report heavy drinking and smoking than urban residents. The results are quite consistent with previous findings from the wide-ranging Chinese populations [2, 8, 10, 31, 34, 35]. In the national level, the behavioral risk factors were highly prevalent in north and northeast areas in China, which were consistent with that from the Sino-MONICA project (1992–1993) [32]. A possible explanation for the geographical variations in behavioral risk factor prevalence in China is that residents in northern China and urban areas may have higher intakes of dietary lipids and alcohol, compared to residents in southern China and rural areas [36]. In addition, unhealthy diets (e.g., high in fat/sodium and low in fruit and vegetables) and increased work-related stress are other lifestyle factors that are associated with urbanization and that are also likely to contribute to the urban-rural differences in the prevalence of obesity and physical inactivity [37]. However, future studies are needed to further clarify to what extent these factors can account for the regional/geographic differences in the prevalence of behavioral risk factors in China. Our findings on overall prevalence and geographically specific prevalence of behavioral risk factors in China serve as a catalyst to enhance linkages among local regions working toward health promotion and chronic disease prevention. This will help to advocate for policy changes at the local, provincial, and national levels and seek funding to support community health promotion and CVD prevention.

The harmful effects of behavioral risk factors on cardiometabolic risk factors have been identified in a series of previous studies [6, 7]. We additionally displayed that the associations between behavioral risk factors and cardiometabolic conditions varied among those who were with cardiometabolic conditions. That is, patients who were aware of their diagnosis of hypertension, high cholesterol, and diabetes were less likely to be current smokers and heavy drinkers, but more likely to be physically inactive and obese. Yet current smoking, heavy drinking, and obesity were associated with the newly detected cardiometabolic cases. The recently released “Health China 2030” blueprint guide has set controlling risk factors as one goal, suggesting the Chinese government should emphasize disease prevention and encourage people to adopt healthy lifestyles [38]. Our results suggest that further improvement in non-pharmacological interventions is needed, such as in exercise and weight control, among patients with cardiometabolic conditions. The previously released Chinese overweight/obesity expert consensus document has provided scientific guidelines on managing factors such as physical activity and diet control for obese/overweight adults, especially patients with chronic diseases to reduce weight [39]. Referring to our results, further efforts are required to improve healthy behaviors in middle-aged and older Chinese adults, and much more efforts should be put in encouraging physical activity and weight control in the primary and secondary prevention of CVDs.

The strengths of this study include its large sample size, nationally representative sampling, a detailed questionnaire and measurements on lifestyle factors, anthropometric measures and blood tests. There were several limitations that should be considered. Firstly, a self-administered questionnaire was used to collect information on smoking status, alcohol consumption and physical activity, and this may have resulted in inaccurate estimations of certain behavioral risk factors. Secondly, the cross-sectional design of this study might limit our possibility to investigate the causal effect of behavioral risk factors and cardiometabolic conditions. Thirdly, certain provinces in China (e.g., Tibet and Ningxia) were missing in this national survey, which limited our ability to estimate the prevalence of behavioral risk factors across all provinces in China. Fourthly, although the distribution of behavioral risk factors in CHARLS 2011–2012 seems quite comparable with the findings observed in the early 1990s [32], the prevalence may differ in the 2020s. Future research is necessary to investigate the trends in the prevalence of behavioral risk factors in China across decades.

## Conclusions

Our study demonstrates that among middle-aged and older Chinese adults, the prevalence of behavioral risk factors varies by age, sex, living areas (rural/urban), and geographic regions. Further effort is required to improve healthy behaviors (e.g. healthy diet and physical exercise) in middle-aged and older Chinese adults, especially in adults with cardiometabolic conditions. This will benefit the primary and secondary prevention of CVDs in this population.

## Supplementary information

**Additional file 1: Supplementary Table 1.** Geographic prevalence (95% confidence interval) of smoking, drinking, obesity and physical inactivity. **Supplementary Fig. 1**. Province-specific prevalence of current smoking, heavy drinking, physical inactivity and obesity by urban and rural areas. **Supplementary Table 2.** Association between behavioral risk factors and cardiometabolic conditions. **Supplementary Table 3.** Association between behavioral risk factors and cardiometabolic conditions among those without history of cardiovascular disease.

## Data Availability

The datasets generated and analyzed during the current study are available in the CHARLS website, available in http://charls.pku.edu.cn/en.

## Abbreviations

CVDs: Cardiovascular Diseases
CHARLS: The China Health and Retirement Longitudinal Study
PSUs: Primary Sampling Units
BMI: Body Mass Index
METs: Metabolic Equivalents
CIs: Confidence Intervals
