# Supplementary material for: Smoking, heavy drinking, physical inactivity, and obesity among middle-aged and older adults in China: cross-sectional findings from the baseline survey of CHARLS 2011–2012
Source: BMC Public Health. 2020 Jul 6;20:1062. doi: 10.1186/s12889-020-08625-5 (PMC7336642; doi:10.1186/s12889-020-08625-5)
Supplement: Supplementary file 1 — Additional file 1: Supplementary Table 1. Geographic prevalence (95% confidence interval) of smoking, drinking, obesity and physical inactivity. Supplementary Fig. 1. Province-specific prevalence of current smoking, heavy drinking, physical inactivity and obesity by urban and rural areas. Supplementary Table 2. Association between behavioral risk factors and cardiometabolic conditions. Supplementary Table 3. Association between behavioral risk factors and cardiometabolic conditions among those without history of cardiovascular disease. [file 12889_2020_8625_MOESM1_ESM.docx]

**SUPPLEMENTARY MATERIALS**

**Supplementary Table 1.** Geographic prevalence (95% confidence interval) of smoking, drinking, obesity and physical inactivity

|  | Participants | Current smoking | Heavy drinking | Physical inactivity^a^ | Obesity |
| --- | --- | --- | --- | --- | --- |
| **Central** | 2705 | 26.74(24.22—29.27) | 6.24(4.76—7.72) | 43.69(37.05—50.34) | 11.14(9.15—13.12) |
| **East** | 5239 | 26.9(25.43—28.37) | 9.06(7.83—10.28) | 43.7(39.11—48.28) | 11.55(9.76—13.34) |
| **North** | 2351 | 29.73(26.35—33.12) | 6.57(4.86—8.27) | 53.07(47.25—58.9) | 19.36(16.39—22.34) |
| **Northeast** | 1288 | 31.12(28.12—34.13) | 9.63(7.31—11.95) | 54.91(46.44—63.37) | 14.19(12.08—16.3) |
| **Northwest** | 1234 | 27.38(23.69—31.07) | 1.19(0.54—1.83) | 38.42(28.98—47.86) | 10.12(7.41—12.83) |
| **South** | 1546 | 23.05(18.22—27.88) | 3.8(0.91—6.68) | 45.67(32.03—59.3) | 8.66(3.57—13.76) |
| **Southwest** | 2939 | 29.29(26.77—31.81) | 9.67(8.00—11.34) | 34.31(28.46—40.16) | 8.02(6.02—10.02) |

^a^Physical inactivity was defined in a subsample, with participants number 6761, of them, 993 was in central, 2078 was in east, 902 was in North, 532 was in Northeast, 471 was in Northwest, 1197 was in South, and 588 was in Southwest.


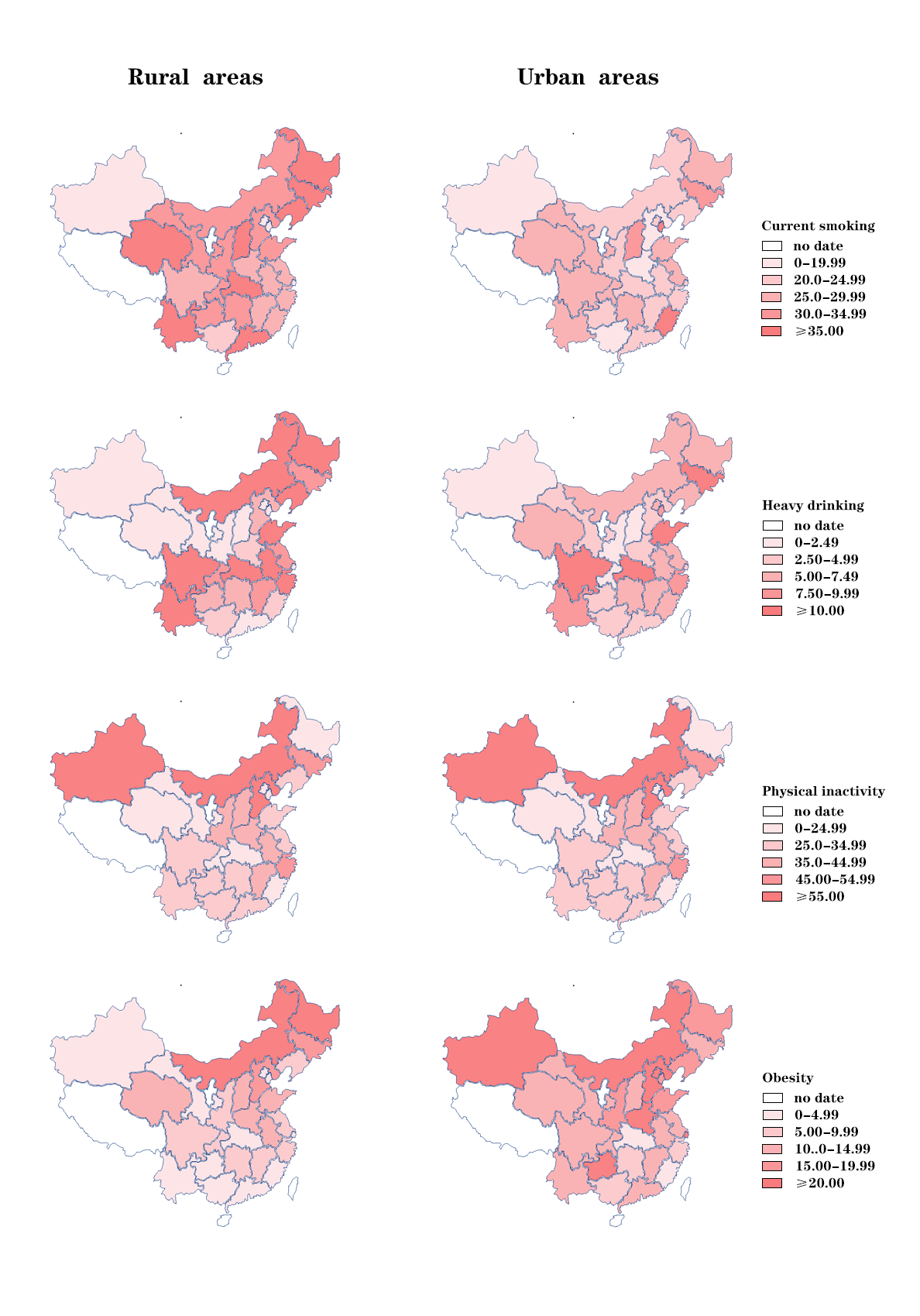


**Supplementary Figure 1**. Province-specific prevalence of current smoking, heavy drinking, physical inactivity and obesity by urban and rural areas

*Notes*. The density maps were generated by DataMap for Excel 5.1.2, using the Chinese geographic map template. An appropriate license from Microsoft has been obtained.

**Supplementary Table 2.** Association between behavioral risk factors and cardiometabolic conditions

|  | **Odds ratio (95% confidence interval)^a^** | | |
| --- | --- | --- | --- |
|  | Hypertension | High cholesterol | Diabetes |
| Current smoking | 0.90(0.78—1.02) | 0.76(0.66—0.88) ^**^ | 0.79(0.64—0.96) ^*^ |
| Heavy drinking | 1.04(0.87—1.24) | 0.95(0.78—1.16) | 0.94(0.74—1.20) |
| Physical inactivity | 1.31(1.15—1.50) ^**^ | 1.31(1.12—1.53) ^**^ | 1.28(1.04—1.56) ^*^ |
| Obesity | 3.40(2.94—3.93) ^**^ | 2.34(1.99—2.75) ^**^ | 1.92(1.59—2.32) ^**^ |

^a^Odds ratios were calculated by combing the newly detected and previous aware groups together as one outcome. Models were adjusted for age, sex, education and rural/urban area.

.^*^0.01<P<0.05; ^**^P<0.01

**Supplementary Table 3.** Association between behavioral risk factors and cardiometabolic conditions among those without history of cardiovascular disease

|  | **Newly detected, Odds ratio (95% confidence interval)** | | |  | **Previously aware, Odds ratio (95% confidence interval)** | | |
| --- | --- | --- | --- | --- | --- | --- | --- |
|  | Hypertension | High cholesterol | Diabetes |  | Hypertension | High cholesterol | Diabetes |
| Current smoking | 1.34(1.08—1.66) ^**^ | 1.00(0.79—1.26) | 1.00(0.80—1.26) |  | 0.71(0.60—0.83)^**^ | 0.74(0.58—0.93) ^**^ | 0.61(0.43—0.86)^**^ |
| Heavy drinking | 1.43(1.14—1.80) ^**^ | 1.30(0.99—1.72) | 1.30(0.98—1.73) |  | 0.90(0.72—1.13) | 0.86(0.64—1.16) | 0.62(0.40—0.97)^*^ |
| Physical inactivity | 1.14(0.85—1.52) | 1.12(0.89—1.41) | 0.96(0.71—1.28) |  | 1.20(0.99—1.46) | 1.20(0.94—1.52) | 1.82(1.32—2.52)^**^ |
| Obesity | 2.00(1.52—2.62) ^**^ | 1.27(0.99—1.63) | 1.62(1.24—2.10) ^**^ |  | 4.64(3.91—5.51)^**^ | 3.52(2.70—4.58)^**^ | 2.11(1.52—2.94)^**^ |

*Notes*. Multinomial logistic regression was used to estimate the odds ratio (95% confidence interval) of newly detected and previously aware cardiometabolic conditions in relation to current smoking, heavy drinking, physical inactivity, and obesity. Models were adjusted for age, sex, education and rural/urban area.^*^0.01<P<0.05; ^**^P<0.01
